# The anti-apoptotic PON2 protein is Wnt/β-catenin-regulated and correlates with radiotherapy resistance in OSCC patients

**DOI:** 10.18632/oncotarget.9013

**Published:** 2016-04-26

**Authors:** Maximilian Krüger, Julianna Amort, Petra Wilgenbus, Johanna P. Helmstädter, Irina Grechowa, Julia Ebert, Stefan Tenzer, Maximilian Moergel, Ines Witte, Sven Horke

**Affiliations:** ^1^ Department of Oral and Maxillofacial Surgery – Plastic Surgery, University Medical Center of the Johannes Gutenberg-University, 55131 Mainz, Germany; ^2^ Department of Pharmacology, University Medical Center of the JGU Mainz, 55131 Mainz, Germany; ^3^ Center for Thrombosis and Hemostasis, University Medical Center of the JGU Mainz, 55131 Mainz, Germany; ^4^ Division of Vascular Surgery, Department of Cardiothoracic and Vascular Surgery, University Medical Center of the Johannes Gutenberg-University, 55131 Mainz, Germany; ^5^ Institute for Immunology, University Medical Center of the Johannes Gutenberg-University, 55131 Mainz, Germany

**Keywords:** paraoxonase-2, Wnt / beta-catenin, leukemia, oral squamous cancer cell, tumor

## Abstract

Aberrant Wnt signaling and control of anti-apoptotic mechanisms are pivotal features in different types of cancer to undergo cell death programs. The intracellular human enzyme Paraoxonase-2 (PON2) is known to have anti-apoptotic properties in leukemia and oral squamous cell cancer (OSCC) cells. However, the distinct regulating pathways are poorly understood. First, we present a so far unknown regulation of PON2 protein expression through the Wnt/GSK3β/β-catenin pathway in leukemia and OSCC cells. This was confirmed via in silico analysis, promoter reporter studies and treatment of multiple cell lines (K562, SCC-4, PCI-13) with different Wnt ligands/inhibitors *in vitro*. Ex vivo analysis of OSCC patients revealed a correlation between PON2 and β-catenin expression in tumor tissue. Higher PON2 expression in OSCC is associated with relapse independently of treatment (e.g. surgery/radio-/chemotherapy). These results emphasize the clinical impact of the newly described regulation of PON2 through Wnt/GSK3β/β-catenin. More importantly, the study revealed the fundamental finding of an overall Wnt/GSK3β/β-catenin dependent regulation of PON2 in different cancers, which was confirmed by systematic and multimethodological approaches. Thus, the herein presented mechanistic insight contributes to a better understanding of tumor specific escape from cell death strategies and suggests PON2 as a new potential biomarker for therapy resistance or as a prognostic tumor marker.

## INTRODUCTION

While cardiovascular diseases cause most mortalities in western societies at this time, cancer develops into a constantly increasing disease due to its age relatedness, leading to an expected rate of almost 1.7 Mio newly diagnosed cancer cases just in the U.S. in 2015 [1]. Although in recent years, several new therapeutics have been developed to treat (or even cure) cancer, there is an increasing need to identify underlying mechanisms for the development of patient-tailored approaches. Various pathophysiological mechanisms have been identified that presuppose malignant transformation, ranging from subcellular levels to the overall tumor microenvironment. Among these, major characteristics of malignancy are sustained proliferative signaling, resistance to target therapies, escape from cell death and activation of invasion and metastasis [2]. The plurality of involved signal pathways reflects the diversity of the different cancer entities. Therefore, it is crucial to gain precise insight into the complex cell death escape strategies of any given tumor in order to develop effective and specific multimodal therapy strategies.

The Wnt signaling pathway has been investigated intensively during the last years because of its dysregulation in different types of cancer. In humans the Wnt family of signaling proteins participates in four signal cascades: the Wnt/β-catenin or canonical pathway, the planar cell polarity pathway, the Ca^2+^/protein kinase A pathway and the pathway involving protein kinase C (PKC), with the canonical pathway being the best studied signal cascade [3]. The central mediator of the canonical pathway is β-catenin (β-cat), which can be activated by several external ligands. Typically, β-cat is kept inactive by a complex consisting of Casein kinase 1 (CK-1), Glycogen-synthase kinase 3β (GSK-3β), Axis inhibitor (Axin) and Adenomatous polyposis coli (APC) [4]. In case of Wnt stimulation, the inactivation of GSK-3β causes a relief of the β-cat-destabilizing phosphorylation, allowing ß-cat to migrate into the nucleus for activation of transcription factors of the T-cell factor (Tcf)/lymphocyte-enhancer-binding factor (Lef) family and adaption of gene expression [5]. However, different combinations of the known 19 ligands paired with up to 10 different receptors give rise to diverse Wnt effects in e.g. proliferation, specification, survival, migration, polarity and even self-renewal of stem cells [6]. As a result, deregulated Wnt signaling makes it a key signal transducing pathway in different types of cancer. Wnt signaling via the canonical and non-canonical pathway was identified to regulate hematopoietic ontogeny in fetal and adult hematopoiesis: Overexpression of Wnt proteins and defective GSK-3β were found to be associated with pre-B-cell leukemia and chronic myeloid leukemia (CML) respectively, while in acute myeloid leukemia (AML) overexpression of β-cat is associated with a poor prognosis [7], [8], [9], [10]. Beside hematological malignancies, a contribution of dysregulated Wnt signaling was also demonstrated for oral squamous cell cancer (OSCC) and aberrant accumulation of β-cat seems to support invasion and migration of OSCC cells, at least *in vitro* (see [11] and references therein). In accordance, Padhi et al. reported of a correlation between β-cat expression and poor prognosis in head and neck cancer [12] *in vivo*. This is supported by the very recent finding that inactivation of several Wnt inhibitors through methylation is frequently found in oral cancer, correlating with a shorter survival [13]. The association of aberrant Wnt signaling to e.g. AML, CML, and OSCC has been demonstrated [14], [15], yet the Wnt-proximal effector molecules remain poorly understood. Therefore, current and upcoming research has to gain more detailed insight in Wnt signaling to identify possible therapeutic targets by pharmacological agents or biologicals. This hypothesis is strengthened by the remarkable finding that treatment of OSCC cell lines with anti-Wnt-1 antibodies diminished proliferation and induced apoptosis by blockade of the canonical pathway [16], [17]. Additionally, beneath aiming on Wnt signaling and its downstream effects directly, superior regulatory signal pathways are attractive targets as well: For instance Funato et al. identified an activation of Wnt signaling through oxidative stress [18], while cellular defense against reactive oxygen species (ROS) is mediated by β-cat [19]. Overwhelming ROS levels have crucial impact on cell signaling, apoptosis, angiogenesis, immune reactions, genetic instability and thereby influence carcinogenesis [20]. Similar to the Wnt pathway, formation of ROS has a central role in carcinogenesis and cancer research, albeit the distinct relationship between Wnt signaling and ROS in cancer and their mutual regulation has not been investigated intensively [21], [22]. Anyway Wnt signaling and ROS are decisive factors for the regulation of molecular cell death programs. This enforced us to investigate the impact of the anti-apoptotic protein Paraoxonase-2 (PON2) on cancer and its possible regulation through Wnt signaling. PON2 is a member of the family of paraoxonases (PON1, PON2 and PON3), localized to the endoplasmatic reticulum (ER) and nucleus [23], [24]. We recently showed an upregulation of PON2 in various solid tumors, as well as in different leukemic diseases [25], [26], [21]. PON2 overexpression raised cancer cell resistance against cytotoxic stimuli, incl. chemotherapeutics, whereas PON2 deficiency enhanced the susceptibility [26], for instance Imatinib-sensitivity of CML-like K562 cells. This evident role in cell death resistance may explain why PON2 levels in pediatric acute lymphatic leukemia (ALL) and chronic myeloic leukemia (CML) were associated with poor prognosis and resistance against Imatinib, respectively [27], [28], [29]. Other *in vitro* studies of our group showed a variable basal PON2 expression in several OSCC cells, with SCC-4 or PCI-13 cells presenting the highest or lowest PON2 levels, respectively. Remarkably, irradiation-induced cell death was much lower in SCC-4 compared to PCI-13, unless PON2 was knocked down by RNAi. Irradiation had the lowest inductive effect on PON2 protein expression in SCC-4, indicating that PON2 counteracts irradiation-induced apoptosis without additional upregulation due to its higher basal expression [30]. These findings prompted us to analyze the *in vivo* PON2 expression in oral cancer and its role in patient' irradiation resistance in a clinical setting.

Taking all this into account and with special regard to aberrant Wnt signaling in cancer, we supposed a regulation of the anti-apoptotic protein PON2 through Wnt signaling in two different cancer sites, which was confirmed in the current study. Our *in vitro* results demonstrate, for the first time, an enhancement of PON2 transcription and translation through Wnt/β-cat mediated Lef-1 activation in leukemia and OSCC cells. More remarkably, the *in vivo* approach unveiled a correlation between PON2 expression and relapse, therapy effectiveness and β-cat levels in OSCC, and points on a so far unknown direct influence of PON2. These results emphasize the clinical relevance of our study: Despite of recent advances in molecular biology of OSCC and the adjuvant therapy strategies, the overall 5-year survival rate of 50% has not been improved during the last decades. Since higher PON2 expression correlates with relapse, our data introduce PON2 as a possible prediction marker for high aggressive OSCC variants resistant to adjuvant treatment modalities e.g. irradiation or chemotherapy. Finally, we believe that the hereby enlightened regulation of the anti-apoptotic PON2 through Wnt/β-cat in cancer justifies the need for further studies and can help to develop new therapeutic strategies in anticancer therapy.

## RESULTS

### Stressed leukemic cells up-regulate PON2

**Figure 1:**
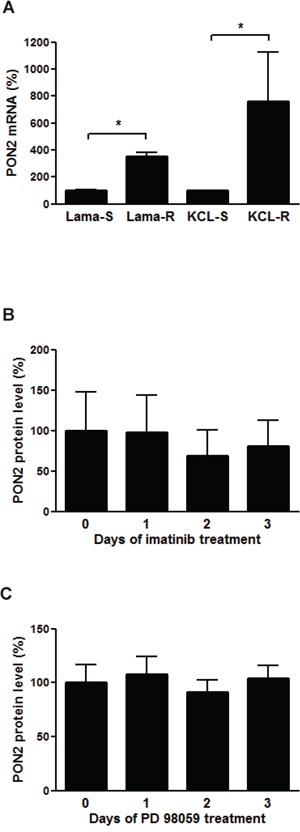
PON2 is highly overexpressed in Imatinib resistant cells, but neither Imatinib nor ERK have a direct effect on the expression **A.** Lama84 and KCL22 cells sensitive (S) or resistant (R) to Imatinib were analyzed for PON2 mRNA levels by qRT-PCR. **B.** K562 cells were treated with Imatinib (0.3 μM) for indicated times. Lysates were analyzed by Western blotting with anti-PON2 and anti–α-tubulin antibodies. **C.** Similar analysis as in B but employing the ERK inhibitor PD 98059 (10 μM). Symbols represent mean ± S.E.M. n = 3 – 13; *P < 0.05.

Previous studies linked PON2 with several diseases including leukemia (see [26] and references therein) and showed that CML-like K562 cells obviously required PON2 for both survival and resistance against the CML chemotherapeutic Imatinib Mesylate (STI-571) [25]. Here, we investigated PON2 expression in additional Bcr/Abl-positive CML cell lines, Lama84 and KCL22, either sensitive (S) or resistant (R) against Imatinib [31]. Both resistant lines Lama84-R and KCL22-R expressed significantly higher PON2 levels (Figure 1A) than their Imatinib-sensitive counterparts. This up-regulation generally resulted from long-term cell adjustment in response to chronic drug exposure, as Imatinib did not alter acute PON2 levels (Figure 1B). Similarly, neither Bcr/Abl activation nor ERK inhibition by PD98059 affected PON2 expression (data not shown / Figure 1C), although ERK is involved in Imatinib resistance. Together with previous results, this verifies the tumor cell-stabilizing effect of PON2 and raises major interest in regulation of its expression.

### Identification of comprehensive PON2 regulation by assay integration

**Figure 2:**
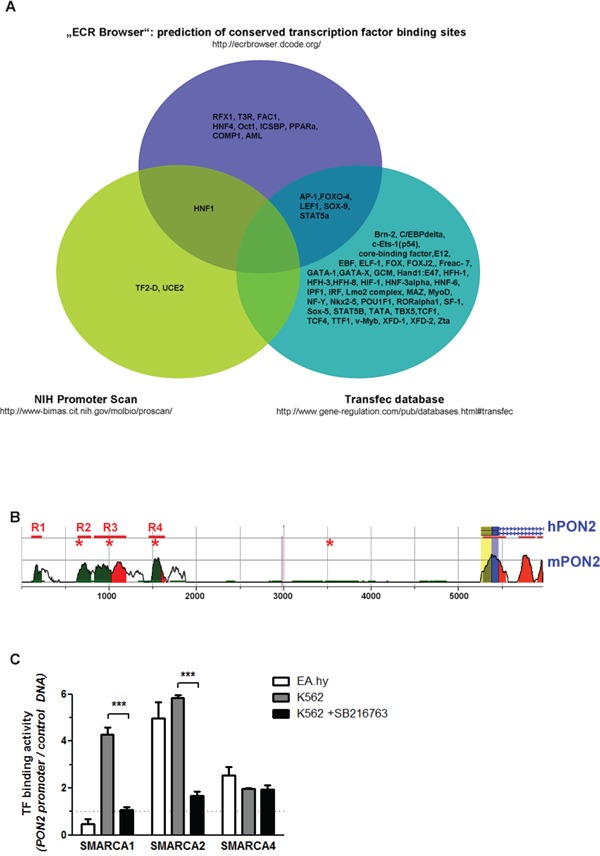
Identification of pathways and / or transcription factors involved in PON2 regulation **A.** The indicated three databases were used for *in silico* prediction of transcription factors (TFs) that bind to a 10-kbp sequence putatively containing PON2-regulating elements upstream transcription start. See text for further details. **B.** Scheme depicting evolutionary conserved regions in human (h) and mouse (m) genomic context upstream PON2 transcription site. Peaks indicate numbers of conserved (above 50%) nucleotides; blue = coding exons; yellow = UTRs; green = transposable elements; red = conserved areas in intergenic regions; R1 – R4 with bars = evolutionary conserved regions; asterisks = Lef-1 sites; blue line with arrows within = protein-coding region. **C.** Control DNA or a 7,5-kbp stretch of human genomic DNA upstream PON2 transcription start was bound to beads and incubated with nuclear extracts isolated from endothelial EA.hy 926 or K562 cells (±SB216763). TFs binding to PON2 promoter but not to control DNA were identified by mass spectrometry employing three independent biological repeats. The graph depicts binding of SMARCA family members.

To uncover relevant pathways and transcription factors (TFs) that may regulate PON2, we first generated a primary collection of potential hits through different approaches. As first approach, using a 10,000 bps sequence stretch originating from the region just upstream of the PON2 transcription start site on human chromosome 7q21.3, we performed three computational searches: (a) putative polymerase-II promoter sequences were identified through PROSCAN search [32]; (b) TF binding-sites were searched by TRANSFAC BIOBASE database (http://www.biobase-international.com); and (c) PON2-regulating TFs were predicted based on binding sites evolutionally conserved between mice and humans through the ECR database [33]. This gave a heterogeneous TF hit list with limited overlap (Figure 2A). Because TRANSFAC and PROSCAN did not identify any common candidate, no TF was projected by all databases. HNF1 was predicted by PROSCAN and ECR. In turn, TRANSFAC and ECR found AP-1, FOXO-4, SOX9, STAT5a and Lef-1. Within the 10 kbp sequence, the ECR database also identified four regions conserved between mice and humans (designated R1 – R4 with a length of 114 – 367 bp in Figure 2B; Supplementary Table S1). Three of these regions (all but R1) contained Lef-1 binding sites. Further, the only evolutionary conserved site is a Lef-1 site in R4 ~3.7 kbp upstream transcription start. Using the Lef-1 consensus site C/TCTTTGAA, hand-search identified a further putative recognition site ~1.7 kbp upstream transcription start in a non-conserved area. Lef-1 is part of the Wnt/β-catenin pathway involved in many of the PON2-associated cancers, including leukemias and thus was the most promising candidate from this approach. In support, TRANSFAC identified further members of Wnt/β-catenin signaling, i.e. TCF1 and TCF4.

For the second method, a 7.5 kbp fragment of the PON2 genomic upstream region was cloned into a pGL4 gene reporter vector. This promoter sequence or an unrelated control DNA were then PCR-amplified, coupled to magnetic beads and incubated with nuclear extracts isolated from endothelial EA.hy 926 cells or leukemic K562 cells. Given aforementioned results, we also used K562 cells pre-treated with the GSK3-β inhibitor SB216763 to activate Wnt/β-catenin signaling (see below). After several washing steps, LC/MS was applied to identify proteins bound to either DNA. Again, this gave a heterogeneous hit list (data not shown) of which we only followed those TFs specific for promoter versus control DNA. Some TFs bound PON2 promoter in a fashion unique to endothelial or leukemic cells, whereas several TFs associated in general and some were changed by SB216763. In particular, this method revealed intriguing results for SMARCA1, SMARCA2 and SMARCA4 (Figure 2C) which belong to the ATP-dependent helicase and chromatin-remodeling SWI/SNF family of TFs. These TFs assemble in a varying fashion to regulate gene expression from a polymorphic interaction surface and they represent attractive drug targets as they appear essential for acute myeloid leukemia maintenance [34]. SMARCA1 bound PON2 promoter DNA just when isolated from K562 and only if Wnt/β-catenin was inactive. SMARCA2 bound PON2 promoter when isolated from both endothelial and K562 cells and its binding was also lost by pre-activation of Wnt/β-catenin. In contrast, endothelial and K562 SMARCA4 associated with PON2 promoter DNA and was independent of SB216763 treatment (Figure 2C). Lef-1 or TCF members were not reliably identified, but this method confirmed a pronounced effect of Wnt/β-catenin signaling on TF binding to PON2 promoter.

As third approach, we used an siRNA array in which we knocked-down 76 major TFs in K562 cells followed by qRT-PCR-based PON2 mRNA quantification. Setting an artificial threshold of an at least 2-fold change, several TFs altered PON2 mRNA (Supplementary Figure S1). This assay confirmed the Wnt/β-catenin signaling factors Lef-1 and TCF4 (=TCF7L2). Intriguingly, their effects opposed each other as Lef-1 deficiency produced a decrease whereas TCF4 deficiency provoked an increase of PON2 mRNA.

Collectively, each approach produced promising candidates, however several hits identified by just one method were not confirmed by other knock-down or functional assays (e.g. Annexin-A2, Vimentin, AUF1, hypoxia / Hif1a, Bcr/Abl, Flt3; data not shown). Thus, we decided to follow hits only when positive in at least 2 out of 3 methods and focused on the Wnt/β-catenin pathway as this was found in every approach.

### Wnt/β-catenin controls PON2 expression through Lef-1 and TCF4-mediated promoter regulation

**Figure 3:**
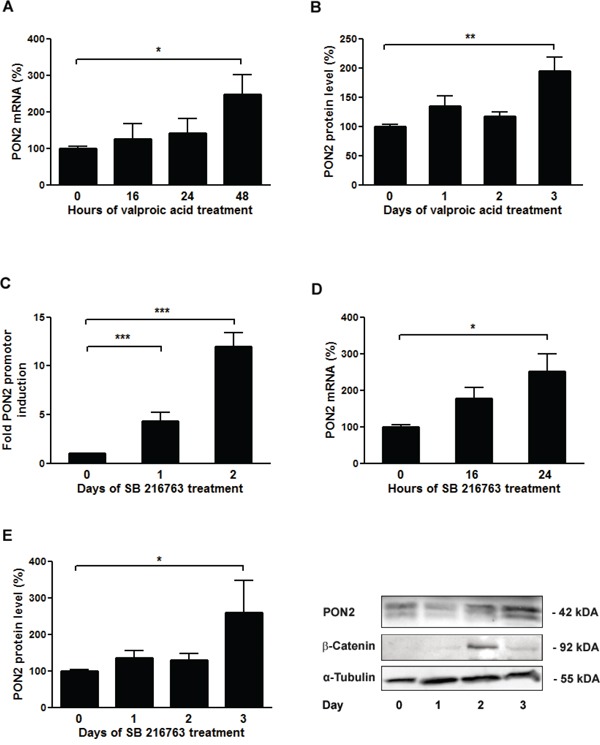
Inhibition of GSK-3β results in up-regulation of PON2 expression in K562 cells K562 cells were treated with valproic acid (1mM) for the indicated times and analyzed for **A.** PON2 mRNA levels by qRT-PCR or **B.** PON2 protein levels by Western blotting. **C.** K562 cells were transfected with a PON2 promoter activity reporter plasmid and were untreated or treated with the GSK-β inhibitor SB216763 (25μM) for the indicated times. Subsequently PON2 promoter induction was quantified. **D**, **E.** K562 cells were treated with SB216763 (25μM) for indicated times and quantified for PON2 mRNA (D) or PON2 and β-catenin protein level (E). One representative blot is shown (right). Symbols represent mean ± S.E.M. n = 4; * P < 0.05; **P < 0.01; ***P < 0.001.

To assess if Wnt/β-catenin signaling influenced cellular PON2 expression, K562 cells were treated with valproic acid (1 mM) for up to three days. PON2 expression was detected at mRNA level by qRT-PCR and at protein level by Western blotting. Valproic acid doubled endogenous PON2 expression after 2 and 3 days at the mRNA and protein level, respectively (Figure 3A, 3B). To add an alternative pharmacologic compound with higher pathway specificity, we switched to SB216763 (25 μM). This compound inhibits the β-catenin-destabilizing kinase GSK3-β and thereby activates β-catenin. Moreover, transcriptional regulation was investigated by inclusion of gene reporter studies for which we transfected K562 cells with the 7.5 kbp promoter reporter plasmid described above. These assays revealed that β-catenin stabilization through SB216763-mediated GSK3-β inhibition induced a PON2 promoter activity with a very pronounced response observed at day 2 (Figure 3C). Alike, PON2 mRNA and protein increased significantly after 2 and 3 days of treatment, respectively. The strongest level of PON2 induction was thus evident at the time of maximal β-catenin stabilization, i.e. after 2 days of treatment (Figure 3D-3F). Although β-catenin levels faded after 3 days, PON2 protein levels stayed high, likely due to a long half-life of its mRNA and protein [35].

**Figure 4:**
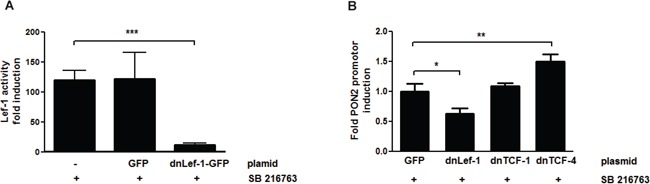
PON2 expression is regulated by LEF-1 and TCF4, but not TCF1 K562 cells were co-transfected with plasmids encoding a firefly luciferase gene under the expressional control of the 7-LEF-fos-luc **A.** or PON2 promoter fragment **B.**, a plasmid for constitutive expression of Renilla luciferase (normalization) and pEGFP-C1, pEGFP-C1-dnLEF-1, pEGFP-C1-TCF1 or pEGFP-C1-TCF4 plasmids. At 4 h after transfection, cells were treated with SB216763 (25 μM) for 24 h. Subsequently 7-LEF-fos-luc or PON2 promoter induction was analyzed, normalized to Renilla luciferase activity and expressed as fold induction. Symbols represent mean ± S.E.M. n = 2; * P < 0.05; **P < 0.01; ***P < 0.001.

Above computational results suggested PON2 regulation by the Wnt/β-catenin pathway, particularly via Lef-1 / TCF transcription factors. To test this in cells, endogenous proteins were inactivated by transfection of plasmids encoding for dominant-negative (dn)-Lef-1, dn-TCF1 or dn-TCF4 proteins. As prior verification of this approach, K562 cells were transfected with a Lef-1 activity reporter gene plasmid and treated with SB216763. This revealed a prominent activation of a Lef-1 reporter gene by SB216763 (Supplementary Figure S2), which was strongly inhibited by expression of the dn-Lef-1 protein when compared to untransfected or GFP-transfected controls (Figure 4A). Thus, dn-Lef-1 largely impairs the activation of endogenous Lef-1 in SB216763-treated K562 cells. We then transfected K562 cells with GFP (control), dn-Lef-1, dn-TCF1, or dn-TCF4 plasmids, treated them with SB216763 and determined PON2 promoter activation by gene reporter assays as before. Confirming above computational and siRNA array results (Figure 2 + S1), Lef-1 inhibition reduced the effect of SB216763 whereas that of TFC4 increased PON2 induction (Figure 4B). Thus, after β-catenin activation, Lef-1 typically up-regulates PON2 while TCF4 down-regulates its promoter activity; TCF1 has no effect.

### Wnt ligands induce PON2 expression

**Figure 5:**
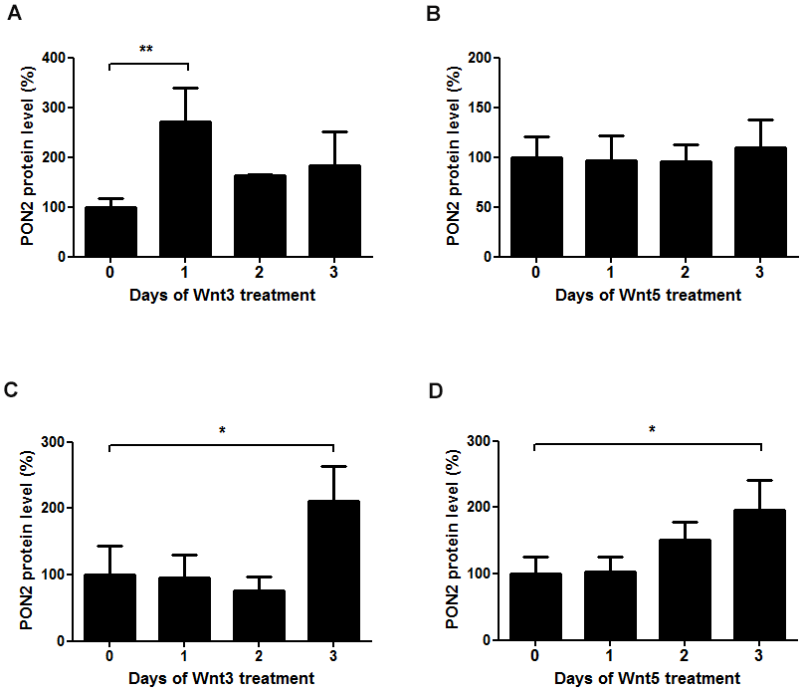
The Wnt ligands 3a and 5a induce PON2 expression **A-B.** Quantitative analysis of PON2 protein expression in K562 cells after treatment with recombinant Wnt3a (100 ng/ml) or Wnt5a (200 ng/ml). PON2 expression was normalized to GAPDH. **C-D.** The same analysis as in A + B, but using primary human umbilical vein endothelial cells (HUVECs). Symbols represent mean ± S.E.M. n = 2 – 5; *P < 0.05; **P < 0.01.

To investigate PON2 induction by upstream Wnt ligands, K562 cells were treated with Wnt3 or Wnt5 for up to three days followed by Western blotting. While Wnt3 led to some increased PON2 expression, Wnt5 had no effect (Figure 5A, 5B). Similarly, Wnt7, Wnt11 or Wnt5+Wnt11 did not change PON2 protein expression during a 3-day period in K562 (not shown). We also tested if these effects were reproducible in non-leukemic cells and treated primary human umbilical vein endothelial cells (HUVECs) with these ligands. Here, Wnt3 but also Wnt5 increased PON2 protein levels (Figure 5C, 5D), which paralleled β-catenin induction in these cells; interestingly, even the non-canonical Wnt5 ligand induced β-catenin in HUVECs (not shown). As in K562 cells, HUVECs did not significantly alter PON2 in response to Wnt7, Wnt11 or Wnt5+Wnt11 (not shown). Thus, our studies demonstrate a Wnt/β-catenin-mediated regulation of PON2 expression in leukemias, a finding backed-up by clinical association studies [25], [26], at least in part.

### Wnt/β-catenin-mediated PON2 regulation in human tumors and cancer cell lines

Next we asked for contribution of Wnt/β-catenin to PON2 regulation in human malignant neoplasms beyond leukemia, because this type of cancer has been investigated before and for the reason that we wanted to test the more general role of this type of PON2 regulation. The Wnt pathway plays a significant role in lung tumors and our previous cell culture experiments demonstrated that lung adenocarcinoma A549 cells, which express high amounts of PON2, went into apoptosis after siRNA-mediated PON2 knock-down [25]. However, the same study reported only low changes of PON2 in (unmatched) lung tumor samples. In support, we here add that quantification of PON2 mRNA levels in patient-matched lung tumor samples again revealed no change in PON2 expression (Supplementary Figure S3), prompting us to not monitor this neoplasm further.

**Figure 6:**
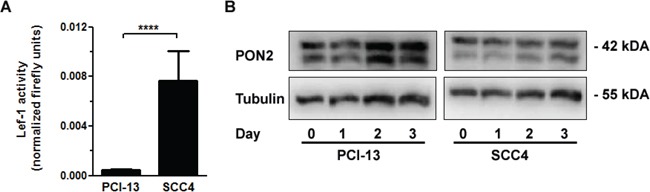
GSK-3β inhibition upregulated PON2 only in oral squamous cancer cell lines with low LEF-1 activity **A.** Oral squamous carcinoma cell lines SCC4 and PCI-13 were transfected with a LEF-1 activity gene reporter plasmid. At 24 h after transfection, LEF-1 promoter induction was analyzed, normalized to Renilla luciferase activity and expressed as fold induction. **B.** PCI-13 and SCC4 cells were treated with SB216763 (25μM) for indicated times and analyzed for PON2 and a-tubulin protein levels by Western blotting. Blots are representative of three others. Symbols represent mean ± S.E.M. n = 3; ****P < 0.0001 (t-test).

We recently assessed the role of PON2 in five human oral squamous cell cancer (OSCC) cell lines. PON2 deficiency increased the cells susceptibility to irradiation; actually the endogenous PON2 expression varied significantly and correlated with resistance against irradiation-triggered cell death [30]. In particular, SCC4 cells with high endogenous PON2 levels were much more irradiation-resistant than PCI-13 cells, which had low PON2 expression. We therefore hypothesized that SCC4 cells with high PON2 levels should have a higher endogenous Lef-1 activity than PCI-13 cells. When both cell lines were transfected with a Lef-1 reporter gene plasmid, we indeed observed a markedly higher endogenous Lef-1 activity in SCC4 compared to PCI-13 (Figure 6A). We thus hypothesized that SCC4 cells have a maximal Lef-1-mediated PON2 induction already at basal levels. Indeed, β-catenin activation through SB216763-treatment was not able to further increase the already high PON2 protein levels. This was in contrast to PCI-13 in which this stimulation led to pronounced PON2 protein up-regulation (Figure 6B).

**Figure 7:**
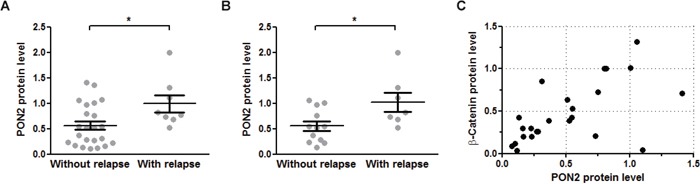
PON2 expression associated with relapse occurrence and β-catenin levels in oral squamous cell tumors **A.** Benign mucosa and tumors were isolated from 32 OSCC patients during tumor-removing surgery and analyzed for PON2 protein level by Western blotting. Patients were grouped as to whether they experienced a relapse during the observation period or not. **B.** Subgroup analysis of the patients from panel A analyzing only those patients that received radio-/chemotherapy after surgical tumor removal. **C.** Subgroup analysis of 24 patients (with or without radio-/chemotherapy) for PON2 and β-catenin protein levels in named samples (tumor / benign mucosa). PON2 and β-catenin expression correlated with Spearman r = 0.8313; 95% CI = 0.6363-0.9265; p<0.0001; Pearson r = 0.7693; 95% CI = 0.5304-0.895; p<0.0001.

Together with our previous study, our findings in OSCC cells suggest that a tumor's PON2 level reflects the cancer cells resistance against irradiation and that PON2 expression may have a predictive value whether or not a post-therapy relapse may occur – in a manner similar to what others showed for PON2 levels in pediatric acute lymphoblastic leukemia [28]. Although a larger trial is beyond the experimental scope of this study, we here assessed PON2 levels in tumors or the normal control mucosa isolated from 32 patients with oral squamous cell cancers. Tumors were removed by surgery and the patients received irradiation (60-70 Gy; in some cases with accompanying chemotherapy) according to their clinical profile. Patients were followed for up to 3 years and monitored for relapse appearance. These studies revealed that PON2 protein levels (determined by Western blotting of matched tumor versus benign mucosa) were significantly higher (p<0.05) in the overall patient group with relapses, compared to those who stayed relapse-free (Figure 7A). This evaluation does not discriminate between patients who received accompanying radiotherapy or not. Moreover, if only patients were taken into consideration who received surgery-mediated tumor removal plus accompanying radiotherapy, PON2 levels remained significantly elevated in the relapse-experiencing group, while it was lower in patients without relapse (Figure 7B). Finally, lysates from a subgroup of 24 patients were assessed by Western blotting and we found a considerable correlation of PON2 and β-catenin protein levels in these samples (tumor / benign mucosa) (Spearman r = 0.8313; 95% CI = 0.6363-0.9265; p<0.0001; Pearson r = 0.7693; 95% CI = 0.5304-0.895; p<0.0001; Figure 7C). Collectively, these findings suggest that in OSCC patients, low PON2 tumor levels predict absence of relapses, whereas higher PON2 levels correlate with elevated β-catenin expression, at least in the majority of cases, and may predict relapse occurrence even after accompanying irradiation therapy.

## DISCUSSION

Cells acquire neoplastic, malignant capabilities by a series of different transformations, one of them being a modulated execution of the cell death program to develop apoptosis resistance. Prior studies have shown that the anti-apoptotic and anti-oxidative enzyme PON2 plays a crucial role in cancer cell survival and that some cancer cells up-regulated PON2 expression by yet unknown mechanisms to benefit from its cytoprotective effect [26], [25], [30]. In particular, pediatric ALL was associated with PON2 overexpression and PON2 was identified as a member of a very small group of upregulated genes that characterized pediatric ALL patients with poor outcome prognosis [28], [27]. Furthermore, we recently showed an involvement of PON2 in radioresistance of OSCC cells [30], yet underlying mechanisms of PON2 gene regulation are incompletely understood. The mechanistic insight in this study provides evidence, for the first time, that PON2 expression in human tumors and cancer cell lines is, at least in part, mediated by the Wnt / GSK-3β / β-catenin pathway. This has been studied for both leukemia models and oral squamous cell cancer.

We here found significantly higher PON2 levels in the Imatinib-resistant CML cell lines Lama84-R and KCL22-R compared to their Imatinib-sensitive counterparts. This fits an earlier report by Frank et al. who described the association of PON2 with Imatinib resistance in CML patients in a BCR-ABL (Philadelphia chromosome)-independent manner [29]. It is known, that BCR–ABL is inhibited by Imatinib and has multiple downstream survival pathways, such as ERK1 / 2, ERK5, AKT, and JAK / STAT (see [36] and references therein). In THP-1 macrophages, Fuhrmann et al. showed an ERK mediated regulation of PON2 expression [37]. Interestingly, short-term treatment of the leukemic cell line K562 with ERK inhibitor PD 98059 or Imatinib has no effect on the PON2 expression. Therefore, it must be assumed that the upregulation of PON2 levels in the resistant cell lines is due to long-term cell adjustment.

To uncover such regulation, we used several methodologically different approaches in the current study, which identified several promising candidates such as LEF-1, TCF4, members of the SMARCA family etc.

As mentioned above, BRM/SMARCA2 and BRG1/SMARCA4 belong to the SWI/SNF chromatin-remodeling complex, which plays essential roles in a variety of cellular processes including differentiation, proliferation and DNA repair [38]. Furthermore, it has been reported that BRM and BRG1 are concomitantly lost in about 15–20% of primary non-small-cell lung cancers [39]. Despite the interesting role of SMARCAs we found only low changes of PON2 in lung tumor / patient-matched NSCLC samples ([25] and Supplementary Figure S3). Therefore, we focused on Wnt/β-catenin as a more promising pathway, since it appeared in every approach, directly or indirectly. Hyperactivation of the Wnt / β-catenin signaling pathway leads to blockade of the kinase GSK-3β and results in a disproportionate activation of proliferative and anti-apoptotic factors, which contribute to the development of various types of cancer [40], [41]. The diversity of pathways with which GSK-3β interacts (e.g. Wnt/β-catenin, PI3K/Akt, Ras/Raf/MEK/ERK and others), makes it a key therapeutic target for leukemia and other diseases [42].

We have validated our hypothesis by demonstrating enhanced PON2 promotor induction, mRNA and protein levels after blockade of the GSK-3β kinase with Valproic acid and the GSK-3β inhibitor SB216763. It has been reported, that stabilized β-catenin enters the nucleus and interacts with transcriptional regulators, including lymphoid enhancing factor-1 (Lef-1) and T cell factors (TCFs), to activate gene transcription [43]. However, studies in *Drosophila melanogaster, Xenopus laevis* and *Caenorhabditis elegans* have indicated that TC factors may also be transcriptional repressors [44]. Based on our knockdown experiments and the usage of dominant-negative isoforms, we were able to identify Lef-1 as a positive and TCF4 as a negative regulator of PON2 expression in the CML cell line K562. Recently, Aly et al. [45] associated high Lef-1 expression with significantly poorer disease-free survival and overall survival in acute lymphoblastic leukemia (ALL) patients. In a similar manner, our findings match earlier studies in which an upregulation of PON2 was identified in a group of patients who suffered from pediatric acute lymphoblastic leukemia (ALL) with poor outcome prognosis [28]. Given that Lef-1 acts as positive regulator of PON2 expression, it will be interesting to study PON2 as potential target or marker in such diseases.

Inhibition of GSK3-mediated β-catenin phosphorylation is the key event in Wnt / β-catenin signaling, but underlying mechanisms remain incompletely understood and it is suspected that GSK-3β exists in three, potentially independent pools sensitive to autonomous stimuli [43]. The Wnt ligands, of which there are at least 16 members in vertebrates, are secreted glycoproteins that can be loosely categorized according to their ability to activate apparently distinct signaling pathways [46], [47]. The verified increase in PON2 expression after administration of Wnt3a and 5a ligands in this study was always correlated with an increase in β-catenin levels. Ligands, which caused no increase in β-catenin, did not alter PON2 expression. This was somewhat unexpected for Wnt5a, since this protein was postulated as a representative ligand that activates β-catenin-independent pathways [48], yet recent studies suggest that the classification of Wnts is too rigid, as individual Wnts can activate both canonical and non-canonical pathways, depending on context [49].

Our prior work demonstrated increased PON2 expression in several tumors and established that selected cells undergo spontaneous apoptosis in response to PON2 knockdown, including A549 lung carcinoma cells [25]. Nevertheless, our current study did not find any change in PON2 expression in different human lung carcinomas. Further, activation of Wnt/β-catenin pathway by GSK-3β inhibition did not change PON2 expression in the OSCC cell line SCC4. Both, human lung carcinomas as well as the SCC4 cell line show high basal PON2 expression. We concluded that a further increase of expression does not occur in already high-expressing tissues or cell lines. In accordance, irradiation enhanced PON2 expression just in low-expressing PCI-13 cells, but not in high-expressing SCC4, despite the fact that both cell lines were susceptible to PON2 knockdown [30]. Therefore, several lines of evidence support a role of PON2 in cancer cell death resistance and a regulation through β-catenin. Importantly, this is corroborated by a marked correlation of PON2 and β-catenin protein levels in tumors of OSCC patients, which in turn could also be linked to relapse occurrence. Importantly, Su et al. recently demonstrated an overexpression of Lef-1 in OSCC samples, which was significantly associated with poor prognosis [50], a finding that may be explained by PON2.

Collectively, our study is the first that systematically addressed endogenous PON2 regulation and how this links to tumorigenesis, demonstrating a significant role for the Wnt–β-catenin–Lef1/TCF axis. Apparently, this appears similarly relevant for both CML and OSCC and may also apply to other forms of malignant cell transformation involving increased β-catenin activity. Based on these findings, targeting PON2 in cancer cells either directly or indirectly through Wnt/β-catenin may contribute to restored death signaling in tumor cells.

## MATERIALS AND METHODS

### Cell culture, transfection and materials

Human tongue squamous cancer cell line SCC-4 and human head and neck squamous cell carcinoma cell line PCI-13 were generously contributed by Professor T. Whiteside, Pittsburgh Cancer Institute. SCC-4, PCI-13, K562, HUVEC and EA.hy 926 cells were cultured as reported earlier [24],[25],[30]. Lama and KCL lines (T. Kindler, Mainz) were cultured under K562-like conditions, but without sodium-pyruvate. For reporter gene assays, we transfect cells with firefly gene reporter constructs and a renilla luciferase encoding control plasmid. Renilla luciferase plasmid was a gift of H. Kleinert (Mainz), plasmids 7-LEF-fos-luc, pEμΔ56 and pcDNA3.1/Zeo(+)-FLAGhdnTCF4 were kind gifts of R. Grosschedl (Max Planck Institute, Freiburg). Activity was assessed with the *Dual-Luciferase*™ Reporter Assay System (Promega). Experiments were performed with 4–8 biological repeats. Where indicated, cells were treated with 25 μM GSK-3β-Inhibitor SB216763, refreshed daily. SB216763 and Valproic acid were from Sigma; Imatinib from Cayman Chemical; Wnt3a/Wnt7a from PeproTech; Wnt5a/−11 from R&D Systems.

### Cloning of plasmids

For cloning a putative 7.5 kb PON2 promoter, we used 500 ng EA.hy926 genomic DNA, 10 pM oligonucleotides (*sense*: 5′-GAGAGGTACCTGTGTGTAGTGAGAGGCAATAGTTC-′3 (KpnI-site); *antisense*: 5′-GAGAGCTAGCCACCTACCTGAGTGCCAGAAG-′3 (NheI-site), 10 nM dNTPs, 6% DMSO, enzyme blend and buffer of Expand-Long-Range-PCR-Kit (Roche, Germany). Using an iCycler (BioRad, Germany), the amplification protocol was (92°C 2min) 1x, (92°C 10sec, 65°C (−1°C/cycle) 15sec, 68°C 7min 45sec) 10x, (92°C 10sec, 55°C 15sec, 68°C 7min 55sec (+22sec/cycle)) 25x, (68°C 7min) 1x. The product represents region −7343 to +114 of human PON2 and was subcloned in the pCR^®^-XL-TOPO^®^-vector with the TOPO®XL-PCR-Cloning-Kit (ThermoFisher Scientific) and further, the promoter was excised by KpnI/NheI (New England Biolabs) and inserted into KpnI/NheI-linearized pGL4.10[luc2] (Promega).

From pEGFP-C1-dnTCF1, dnTCF1 cDNA was NheI/ApaI-excised from pcDNA3.1/Zeo(+)-FLAGhdnTCF4 and cloned into pEGFP-C1 (dnTCF1) or subcloned into pEGFP-N1 (Clonetech) (dnTCF4). For amplification of dnTCF4-cDNA we used 10 pM oligonucleotides (sense: 5′GAGACTCGAGCCGACTACAAAGACGATGACGATAAAAAGGATCC-3′ (XhoI-site); antisense: 5′-GAGAGGTACCTTAGGCCCCGTTGGGACAGAGGGCGGAGGCCTTGTGG-3′ (KpnI-site), 500 ng DNA, dNTPs (10 nM), 2.5 U Taq enzyme and buffer (Roche, Germany). Using an iCycler, the protocol was (95°C 2min) 1x, (95°C 30sec, 55°C 30sec, 72°C 1min 30sec) 35x, (72°C 10min) 1x, followed by cloning into XhoI/KpnI-linearized pEGFP-C1-plasmid.

To generate pEGFP-C1-dnLEF-1, dnLEF-1 cDNA was BglII/EcoRI-excised from pEμΔ56 plasmid and inserted into pEGFP-C1.

### qRT-PCR

Quantitative real-time polymerase chain reaction was performed as reported previously [25]. Taqman primers (Eurofins MWG Operon) were as follows: GAPDH: sense 5′-CAACAGCCTCAAGATCATCAGC-3′; antisense 5′-TGGCATGGACTGTGGTCATGAG-3′; probe 5′-CCTGGCCAAGGTCATCCATGACAAC-3′; mATPSy6: sense 5′-CAGTGATTATAGGCTTTCGCTCTAA-3′ antisense 5′-GGCCAGGGCTATTGGTTGAA-3′ probe 5′-CCCTAGCCCACTTCTTACCACAA-3′; PON2: sense 5′-TCGTGTATGACCCGAACAATCC-3′; antisense 5′-AACTGTAGTCACTGTAGGCTTCTC-3′; probe 5′-TCGTCAGAGGTTCTCCGCATCCAGA-3′; PolR2a primers were from Applied Biosystems (ID. HS 00172187). The amplification protocol was (95°C 1min) 1x, (95°C 1min 30sec, 60°C 30sec) 45x. Relative expression was calculated by the 2[−ΔΔC(T)] method and PON2 normalized to GAPDH, mATPsy6 and/or PolR2a.

### Western blotting and immunodetection

SDS-PAGEs, electrophoretic transfer of proteins, and Western blotting were done as described earlier [35],[24] with antibodies mouse-anti-glycerin-aldehyde-3-phosphate-dehydrogenase (GAPDH) 6C5 from Santa Cruz; rabbit-anti-PON2 [24]; mouse-anti-α-Tubulin Ab2 from Dianova; rabbit-anti-β-Catenin (6B3) from Cell Signaling; HRP-conjugated secondary antibodies from Sigma or Cell Signaling.

### Transcription factor knockdown analysis

To analyse the PON2 regulating pathways in cancer cells we used the reverse-genetic method of the Biology-on-Array Cancer TF siRNA System (SABiosciences/Qiagen) on K562-cells, following the manufacturer's instructions.

### In silico analysis

Following public databases were used: ECR Browser [33]: http://ecrbrowser.dcode.org; NIH Promoter Scan: http://www-bimas.cit.nih.gov/molbio/proscan/; Transfac^®^ database: http://www.gene-regulation.com/pub/databases.html#transfec. For this purpose, the putative PON2 promoter sequence (10,000 bps upstream transcription start) was entered.

### Quantitative proteomic analyses

Cell pellets (three biological replicates) were solubilized in lysis buffer (7 M urea, 2 M thiourea, 5 mM DTT, 2% CHAPS) by sonication for 10 min at 4°C. Tryptic digestion was performed by modified FASP-protocol [51]. Tryptic peptides were lyophilized and dissolved in 0.1% formic acid and spiked with 20 fmol/μL of yeast enolase 1 MassPREP™ protein digestion standard (Waters) prior to LC-MS.

Tryptic peptides were analyzed using a nanoscale UPLC system (nanoAcquityUPLC (Waters)) coupled online to a Synapt G2-S HDMS mass spectrometer (Waters). Peptides were separated on a HSS-T3 1.7 μm, 75 μm x 150 mm reversed-phase column (Waters) using direct injection mode as described before [52]. Analysis was performed in positive mode ESI-MS using MS^E^ in combination with on-line ion-mobility separation (UDMS^E^) as described in detail by Distler et al. [51]. The data were post-acquisition lock mass corrected using [Glu1]-Fibrinopeptide B. LC-MS data were processed using ProteinLynxGlobalSERVER V3.0.2 (PLGS, Waters Corporation) searching against the UniprotKB/Swissprot human database (UniProtKB release 2012_07), which was concatenated to a reversed decoy database, using the following search criteria for peptide identification: i) trypsin as digestion enzyme ii) up to two missed cleavages allowed iii) fixed carbamidomethylcysteine and variable methionine oxidation modifications. Precursor and fragment ion mass tolerances were automatically determined by PLGS3.0.2, resulting in mass tolerances <5 ppm (3.3 ppm RMS) for precursor and <10 ppm for fragment ions. The initial false discovery rate (FDR) for protein identification was 1% in PLGS based on a reversed decoy database search. Data post-processing was performed using the software package ISOQuant, including retention time alignment, exact-mass-retention-time and ion-mobility clustering, signal annotation, normalization and protein isoform/homology filtering [52]. Absolute in-sample amounts were calculated in ISOQuant for each protein based on the TOP3 approach [53].

### Treatment of human specimen

Tumor specimen and benign mucosa samples were taken from 32 patients with OSCC, who underwent tumor resection in the department of oral and maxillofacial surgery – plastic surgery of the University Medical Center Mainz. Harvesting and sample treatment was described previously [30]. Patient consent for obtaining tumor and mucosa specimen was received and an ethics committee approved use of excess material.

### Software, statistics and image acquisition

Device-specific software provided by the suppliers was used. GraphPad Prism 5 software was used for statistical analysis using 2-tailed Student's t test (for normally distributed data, skewness<1) or Mann-Whitney test (for non-normally distributed data, skewness>1), unless otherwise indicated. For analyses of more than 2 groups, 1-way-ANOVA with Bonferroni's multiple comparisons post test was used. Cell culture experiments consisted of 3 or more biologic repeats as indicated in the figures. Data are shown as mean ± SEM. P<0.05 was considered significant.

## SUPPLEMENTARY FIGURES AND TABLES




