# Supplementary material for: The anti-apoptotic PON2 protein is Wnt/β-catenin-regulated and correlates with radiotherapy resistance in OSCC patients
Source: Oncotarget. 2016 Apr 26;7(32):51082–95. doi: 10.18632/oncotarget.9013 (PMC5239460; doi:10.18632/oncotarget.9013)
Supplement: Supplementary file 1 [file oncotarget-07-51082-s001.pdf]

The anti-apoptotic PON2 protein is Wnt/β-catenin-regulated and correlates with radiotherapy resistance in OSCC patients

SUPPLEMENTARY FIGURES AND TABLE

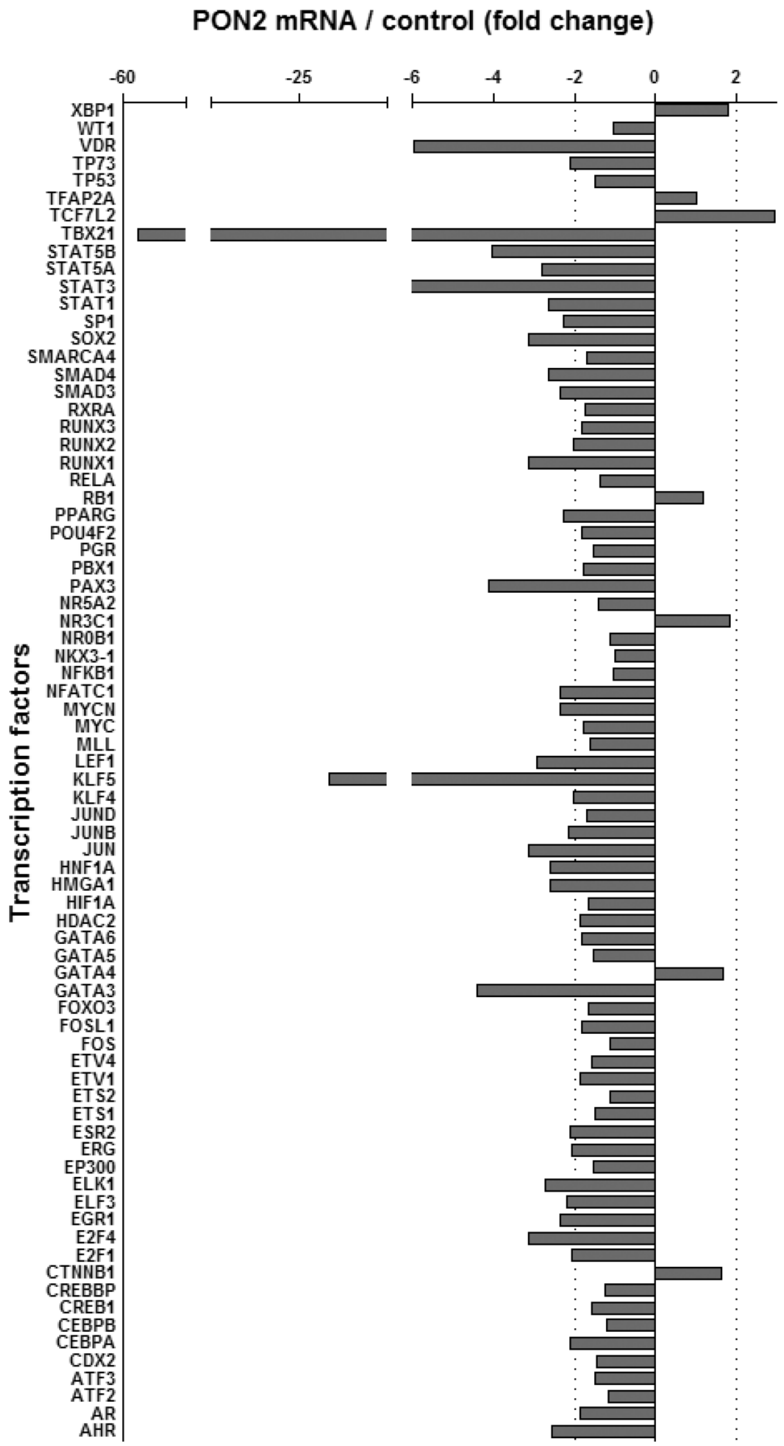

Supplementary Figure S1: Identification of PON2 regulating transcription factors (TF) by knockdown analysis. siRNA array of 76 TF in K562 cells. 3 days after transfection PON2 mRNA levels were quantified by qRT-PCR and normalized to GAPDH, mitochondrial ATP-Synthase 6 (mATPSy6) and RNA-Polymerase 2 (PolR2a).

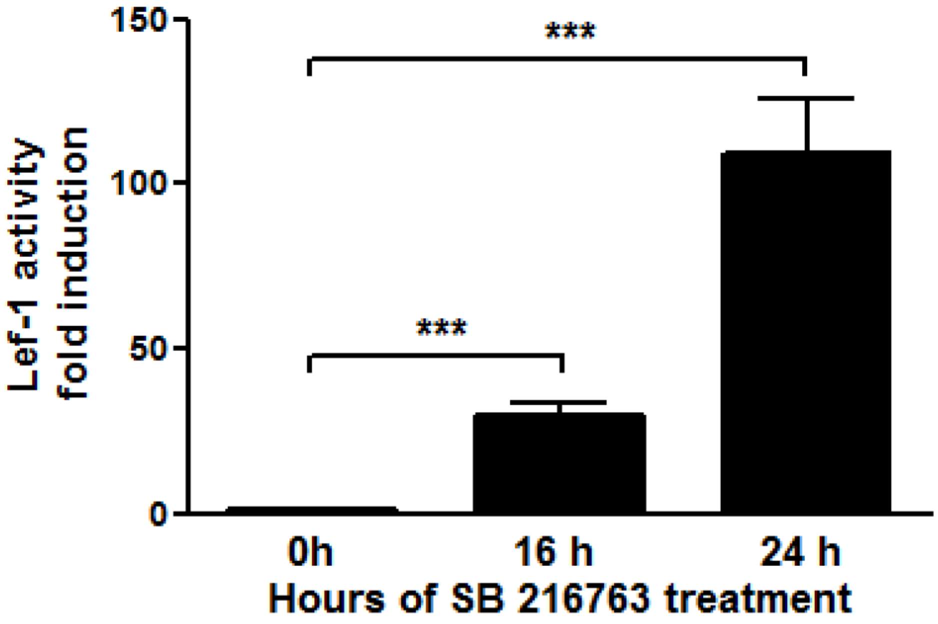

Supplementary Figure S2: Induction of Lef-1 by GSK-3β inhibition. K562 cells were co-transfected with plasmids encoding a firefly luciferase gene under the expressional control of the 7-LEF-fos-luc and a plasmid for constitutive expression of Renilla luciferase (normalization). At 4 h after transfection, cells were treated with SB216763 (25 µM) for indicated times. Subsequently 7-LEF-fos-luc promoter induction was analyzed, normalized to Renilla luciferase activity and expressed as fold induction. Symbols represent mean ± S.E.M. n = 2; ***P < 0.001.

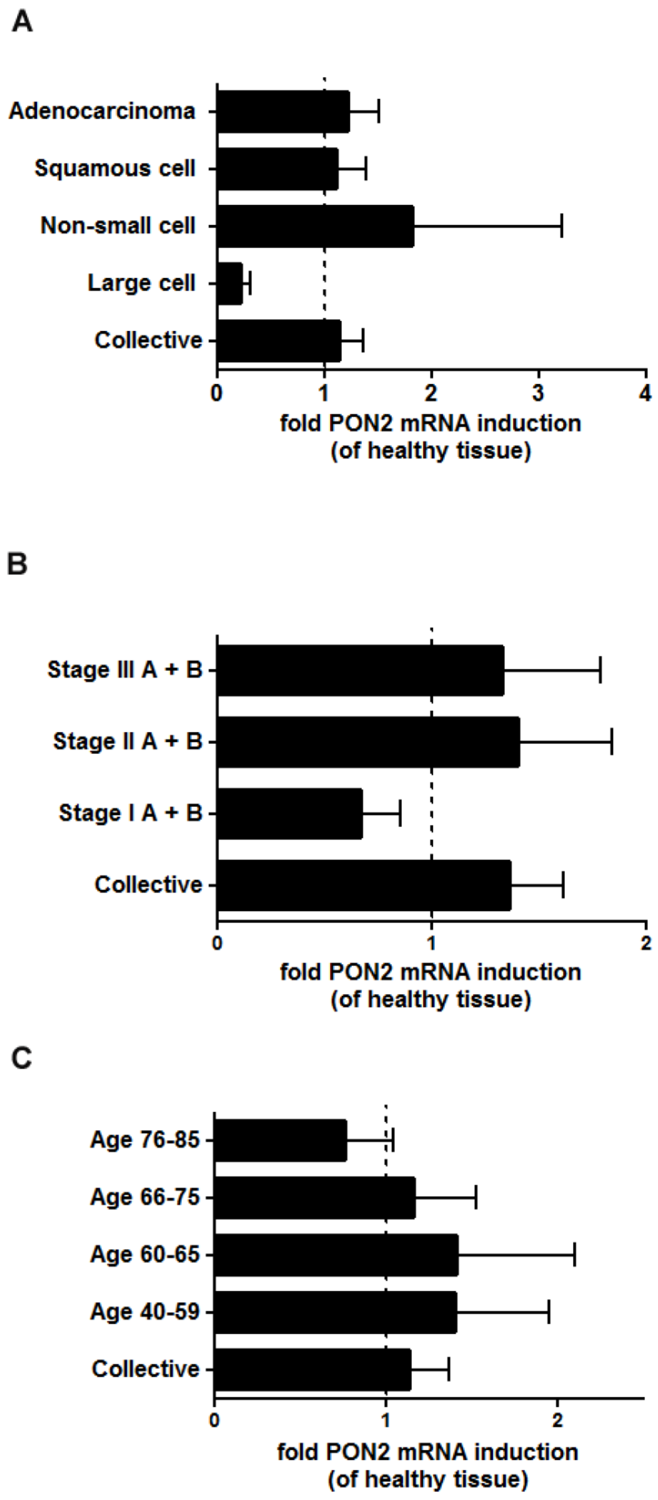

Supplementary Figure S3: PON2 expression levels are unchanged in patients suffering from lung carcinoma. PON2 mRNA expression levels were determined in 24 matched human lung cancer samples versus normal tissues from the same patient by qRT-PCR using TissueScan™ matched Lung Cancer Panel-IV array (Origene, Rockville, MD, USA) following the manufacturer's instructions and normalized to GAPDH. The 24 samples were either summarized ('collective') or artificially separated into different groups of **A.** diagnosis, **B.** stage or **C.** age.

Supplementary Table S1:

See Supplementary File 1
